# Improved Sobriety Rates After Brain-Computer Interface-Based Cognitive Remediation Training

**DOI:** 10.7759/cureus.21429

**Published:** 2022-01-19

**Authors:** Curtis T Cripe, Peter Mikulecky, Michel Sucher, Jason H Huang, Dallas Hack

**Affiliations:** 1 Graduate School of Social Service, Fordham University, New York City, USA; 2 Behavioral Medicine, NTL Group, Scottsdale, USA; 3 Brain Mapping and Optimization, Neurologics, Inc., Newport Beach, USA; 4 Substance Use and Addiction, Meadows Behavioral Healthcare, Phoenix, USA; 5 Neurosurgery, Baylor Scott & White Medical Center, Temple, USA

**Keywords:** quantitative electroencephalography, brain mapping, brain-computer interface, addiction recovery, cognitive remediation therapy

## Abstract

Up to 80% of individuals seeking treatment fail in their attempts at sobriety. This study investigated whether 1) a cognitive remediation therapy (CRT) program augmented with a brain-computer interface (BCI) to influence brain performance metrics would increase participants' self-agency by restoring cognitive control performance; and 2) that ability increase would produce increased sobriety rates, greater than published treatment rates. The study employed a retrospective chart review structured to replicate a switching replication methodology (i.e., waitlist group) using a pre-test and post-test profile analysis quasi-experimental design. Participants' records were organized into treatment and non-treatment groups. Adult poly-substance users were recruited from alcohol and other drugs (AOD) use outpatient programs and AOD use treatment centers in the United States. Participants volunteered for pre- and post-testing without treatment (*n* = 121) or chose to enter the treatment program (*n* = 200). The treatment group engaged in a 48-session BCI/CRT augmented treatment program. Pre- and post-treatment measures comprised 14 areas from the Woodcock-Johnson Cognitive Abilities III Assessment Battery. An 18-month follow-up assessment measured maintenance of sobriety. After testing the difference for all variables across time between test groups, a significant multivariate effect was found. In addition, at 18 months post-treatment, 89% of the treatment group maintained sobriety, compared to 31% of the non-treatment group. Consistent with addiction neurobehavioral imbalance models, traditional treatment programs augmented with BCI/CRT training, focused on improving cognitive control abilities, may strengthen self-control and improve sobriety rates.

## Introduction

Alcohol and other drugs (AOD) dependence and its associated mental health disorders are among the most severe health, economic, and social problems facing the United States [1]. According to the WHO, the cost of AOD dependence worldwide is in the trillions of dollars, with an estimate of over $700 billion in the United States alone [1]. However, the economic costs are not the only costs involved. Social ramifications are significant when families are torn apart. AOD use adversely affects children, spouses, parents, relatives, and other relationships.

According to the National Institute on Drug Abuse (NIDA), addiction is a chronic condition characterized by compulsive cravings, drug-seeking, and drug use that persist despite adverse consequences. Moreover, addiction can reoccur after long periods of abstinence [2-4]. Addiction is a natural, neural adaptation process consequential to drug use, resulting in an inability to make mature decisions regarding drug use, and requires repeated and persistent treatment [2-4]. Although overcoming substance use is one goal of therapy, returning people to productive functioning within the family, workplace, and community is a more compelling and longer-lasting goal.

Meta-analyses of AOD treatment program outcomes report that the average short-term abstinence rates are 20% for untreated individuals, compared with 40% for treated individuals [2-4]. Overall, these reports suggest that treated individuals achieve higher short-term remission rates than untreated individuals. However, these figures also indicate that 60%-80% of individuals who seek treatment fail in their quest to maintain sobriety. Current AOD treatment models address addictive behaviors with a wide range of treatment modalities, including different forms of psycho-education, traditional therapy, pharmacology, 12-step recovery programs, or some combination therein. However, outcome reports that include treatments focused on brain recovery or actual brain repair of self-regulation abilities are absent from addiction treatment literature; lacuna is the focus of this study.

Neurobiological models of addiction seek to broaden the understanding of addiction as a brain disease. These models integrate classic psychological models (such as dual-process theory) with neurobiological responses. According to dual-process theory, individuals learn social rules, which are handled by a reflective system in the brain to control impulsive responses [5-10]. Dual-process theory research suggests that addictive behavior results from an imbalance between two independent, interacting neural systems that control decision making. These systems include a reflexive or automatic system used in signaling immediate pain or pleasure responses (i.e., the reward motivation system) mediated by mesolimbic dopamine circuitry and a reflective system used to evaluate the long-term choice effects (i.e., the executive control system) which is located in the prefrontal and parietal networks [5-10].

According to the dual-process theory, vulnerabilities in these two systems contribute to the development and maintenance of AOD addiction behaviors [8]. For example, the brain's neural focus on high levels of reward motivation likely increases one's inclination toward drug experimentation/use, whereas weakness in executive control is related to the progression of AOD use and compulsive forms of drug use [8-12]. This process is understood to occur in the following sequence: 1) AOD use desensitizes the brain's reward circuits, dampening the ability to feel pleasure and reducing the motivation to pursue everyday activities; 2) conditioned responses to AOD use and stress reactivity increase, which increases cravings for AOD and negative emotions when these cravings are not sated; and 3) brain regions involved in executive functions (e.g., decision making, inhibitory control, and self-regulation) weaken. In combination, this neurobehavioral imbalance/progression leads to repeated relapse. From a neurological systems perspective, this process likely results from a hypoactive prefrontal-mediated executive control system that fails to adequately control a hyperactive striatal reward system [10-12]. In an attempt to confirm this premise, Khurana A et al. compared youths with high impulsivity and sensation-seeking characteristics to those with high and low executive control abilities [13]. They found that weak executive control and heightened reward-seeking predicted the early progression of drug use. Conversely, increased reward-seeking, balanced by a strong executive control system, predicted only occasional experimentation [13].

Implications for breaking the relapse cycle

Individuals early in the recovery process are faced with multiple situations every day in which they must choose to remain sober. Many of these situations compel addicts to maintain a strong sense of self-agency to break non-sobriety supporting habits. As Khurana A et al. reported, habit-breaking abilities require not only good intentions but also robust and resilient cognitive functioning to exercise cognitive control and cultivate new sobriety behavioral habits [13].

According to Chatham CH et al., habit-breaking skills are acquired by progressing through four transitional stages in which individuals learn new skills and then integrate those skills into their daily lives [14,15]. To successfully break the relapse cycle, recovering individuals must effectively navigate through all the transitional stages of recovery. Thus, one must not only intend to remain sober and understand the environmental context of the relapse cycle but also be cognitively equipped to exercise volitional control when that control is needed. Each transitional stage requires the recruitment of different sets of cognitive functions to acquire and execute new skills. The rate of learning, the ability to retain a new skill, and the execution of this skill depend on the learner's health and the functional strength of his or her cognitive function. Unfortunately, many cognitive functions are significantly compromised for many individuals in AOD addiction recovery [14-17].

The capacity and performance of an individual's executive control capabilities dynamically vary in the moment, based on one's current cognitive load, stress level, and resilience to stress [17-20]. For individuals in recovery, low capacity and low stress-resilient cognitive function increase the risk of making poor decisions. As supported by dual-process theory and as evidenced by addiction studies [14-17], unless the individual in recovery can maintain strong reflective abilities (including the abilities to learn, integrate, and self-monitor) and has the neural resilience to withstand daily stress, that individual will remain at risk for relapse. From a brain perspective, the functional strength, health, and ability of the executive control functions are critical to ongoing success.

Strengthening cognitive control abilities

Cognitive remediation therapies (CRTs) fall within the class of cognition-based strengthening interventions [20-22]. Many forms of CRT interventions have been applied successfully among individuals with acquired CNS disorders, including traumatic brain injury, stroke, mental health issues, depression, substance use, and neurodegenerative conditions [20-22]. The brain-behavior relationship and the mechanisms of injury, disease, and recovery inform these therapies. Such interventions reflect two broad conceptual frameworks of functional brain recovery: compensatory and restorative approaches [22,23]. Compensatory interventions focus on translating underlying neuropsychological impairments into environmental adaptations, thereby enabling participation in daily life. The primary goal of compensatory approaches is to help individuals achieve real-world objectives and participate in activities that might be blocked by unrecoverable cognitive impairments. Conversely, restorative approaches use repetitive exercises, similar to the exercises in standardized cognitive abilities tests, to restore dysfunctional cognitive functions (e.g., attention, organization, memory, reasoning, and problem solving). Restorative CRT strengthens underlying neuropsychological impairments located within the brain rather than teaching compensatory or adaptive skills [22,23]. Increased brain activation likely occurs by a progression of synaptic growth and repair generated by repeated practice or the stimulation of specific neuropathways. Supporting evidence for this approach includes a recent functional MRI (fMRI) study that exhibited increased memory-related brain activation following cognitive training in several brain regions in individuals at high risk for dementia due to mild cognitive impairment (MCI) [22,23]. The restorative methods used in this study have been applied successfully to patients with schizophrenia, substance use, or brain injuries, children and adults with ADHD, and for the cognitive deficits associated with major depression [21-23].

## Materials and methods

The study design employed a retrospective chart review methodology to formulate results derived from participants who had previously participated in a brain-computer interface (BCI)-augmented CRT program as a component of their AOD use recovery treatment program. In addition, this study used a profile analysis quasi-experimental design, using participant retrospective records arranged into two non-randomized groups: control and treatment, to explore treatment effects. Participant records were structured with dependent pre- and post-test sampling in both groups. Profile analysis is an application of a multivariate analysis of variance (MANOVA) in which several dependent variables (DVs) are measured on the same scale [24,25], with the more common application where subjects are measured repeatedly on the same DV. Profile analysis offers a multivariate alternative to the univariate F test for the within-subjects effect and its interactions. The analysis asks if the two groups have the same pattern of means on the subscales. Both treatment and non-treatment group participants were concurrently involved in some form of traditional addiction recovery therapy, either through a residential treatment center or an outpatient program. Each participant in the treatment group received an individualized program designed to address neurobehavioral imbalances in their executive function. Targeted treatment variables focused on remediating deficiencies observed in participants' cognitive control, memory, attention, and executive function. Neurobehavioral imbalances were addressed using an advanced form of a CRT employing a BCI method to influence CRT training activities based on the cognitive information processing strength of each imbalance in real-time [15, 21, 26].

Participants

Participants were adults (aged 18 or older), poly-substance users recruited from AOD use outpatient programs and AOD use treatment centers across the United States. Data were collected from 2012 to 2016, with follow-up data regarding maintenance of sobriety collected through 2018. All records were de-identified to protect the anonymity of individual health information. By request of the treatment centers from which participants were recruited, the records of participants were included only when individuals had accrued a minimum of 60 days of sobriety for the treatment group, with 120 days or more for the waitlist group. The participants had been poly-substance users for an average of 17 years and had an average of 10 residential treatment program failures. Participants were matched with regard to age, education, and gender. Treatment and non-treatment group record selection was based on a deliberate self-selection convenient sample method in which participants either volunteered for pre- and post-testing without treatment or chose to enter the treatment program. The treatment group was tested before treatment and upon treatment completion. The treatment group was composed of 200 participant records (n = 200; 100 males and 100 females); the non-treatment comparison group included 121 records (n = 121; 61 males and 60 females). The following exclusion criteria were used for all groups: 1) <60 days of sobriety; 2) a history of severe traumatic brain injury with a loss of consciousness of >30 minutes; and 3) histories of schizophrenia, bipolar disorder, or obsessive-compulsive disorder. All participants provided written consent to participate in the study. Participants' records were divided into a non-treatment group and a treatment group. Each group received the same pre-test.

Experimental pre- and post-test measures

To support a profile analysis of the effect of treatment status (no treatment or treatment) on cognitive ability, participants were measured on 14 subtests of the Woodcock-Johnson Test of Cognitive Abilities III (WJIII) [27]. The WJIII is a set of cognitive ability subtests based on the Cattell-Horn-Carroll theory (CHC) of cognitive abilities. The CHC theory provides a comprehensive framework for understanding the structure of cognitive information processing abilities. The 14 subtest areas were: iQT (fluid intelligence), thinking efficiency, concept formation, working memory, numbers reversed, visual-auditory learning, visual-auditory learning-delayed, verbal ability, phonemic awareness, verbal comprehension, incomplete words, sound blending, spatial relationships, and visual matching. The grouping variable was for BCI/CRT treatment vs. non-treatment.

Tracking sobriety and social reintegration rates

For this study, sobriety was defined as maintaining abstinence from any form of substance use. Social reintegration was defined as maintaining financial independence (i.e., living on one's own and working to support oneself by living independently or being in school). The records of random sets of treatment (n = 50) and non-treatment (n = 50) participants at 18-month follow-up interviews were reviewed to track the integrative effect of the program. In addition, answers to three questions were recorded: 1) How long participants had maintained sobriety? 2) What is the status of their current living situation? and 3) What is their work status?

Procedure and training

The CRT training method used in this study was implemented through a set of training tools composed of a collection of working memory and executive function activities, routinely employed by the primary author in clinical settings to address brain-based deficiencies, called the NeuroCoach program (NTLGroup Inc., Scottsdale) by clients and staff [21, 26]. Each activity was designed to develop cognitive functional capacity within a chosen cognitive ability (e.g., auditory working memory capacity, impulse control on go/no-go tasks, or cognitive flexibility with variations of modified Stroop activities) and to develop resilience when encountering stress. Resiliency was enhanced by demanding greater performance under a larger, more demanding cognitive load based on varying working memory load demands and performance in conjunction with changing response time constraints. In addition, an EEG BCI interface was used to monitor and adjust cognitive loads based on previously identified EEG protocols of addictive drive mechanisms and working memory cognitive load, both of which were used to influence activity presentation [21, 26, 28, 29].

Participants sat in front of a computer screen and performed tasks derived from the WJIII battery, presented by the EventIDE task management program (OkazoLab, Delft, The Netherlands). Each participant performed tasks attached to a 19-channel EEG monitor (impedance below 5 kOhms). Sensors were placed at positions FP1, FP2, F3, Fz, F4, F7, F8, C3, C4, Cz, T3, T4, P3, Pz, P4, T6, T8, O1, and O2, using a BrainMaster 24E acquisition system (BrainMaster Technologies, Bedford, OH) with sampling at 256 Hz. Artifacts detection and removal were performed using the artifact subspace reconstruction (ASR) artifact algorithm (EEGLAB; Swartz Center for Computational Neuroscience, San Diego, CA). Neural metric measures were computed from the preprocessed EEG data using Independent component analysis (ICA)/principal component analysis (PCA) methods. Testing began with a collection of resting-state, eyes-closed and eyes-open conditions as baseline measures. Next, classical, age-normed neurometrics were obtained based on standardized measures of resting-state quantitative EEG (qEEG).

All training group participants completed 48 extensive training sessions (approximately 30-40 minutes per session) before re-evaluation. Immediately after the initial evaluation, the training group used the remediation program three times per week for eight weeks (approximately 30-40 minutes per session); these participants were then reassessed. The training group participated in their traditional addiction therapy program provided by a residential treatment center or outpatient program. The non-training group did not participate in the remediation program but continued with traditional addiction therapy provided by the residential treatment center or outpatient program.

## Results

The mean age of participants was 34 years old and ranged from 24 to 44 years old. Group means were used for data screening. All participants had complete data sets (i.e., no missing data). No univariate or multivariate outliers were detected, with p = 0.001, and assumptions regarding normality of sampling distributions, homogeneity of variance, covariance matrices, linearity, and multicollinearity were met.

Effects on cognitive abilities

**Figure 1:**
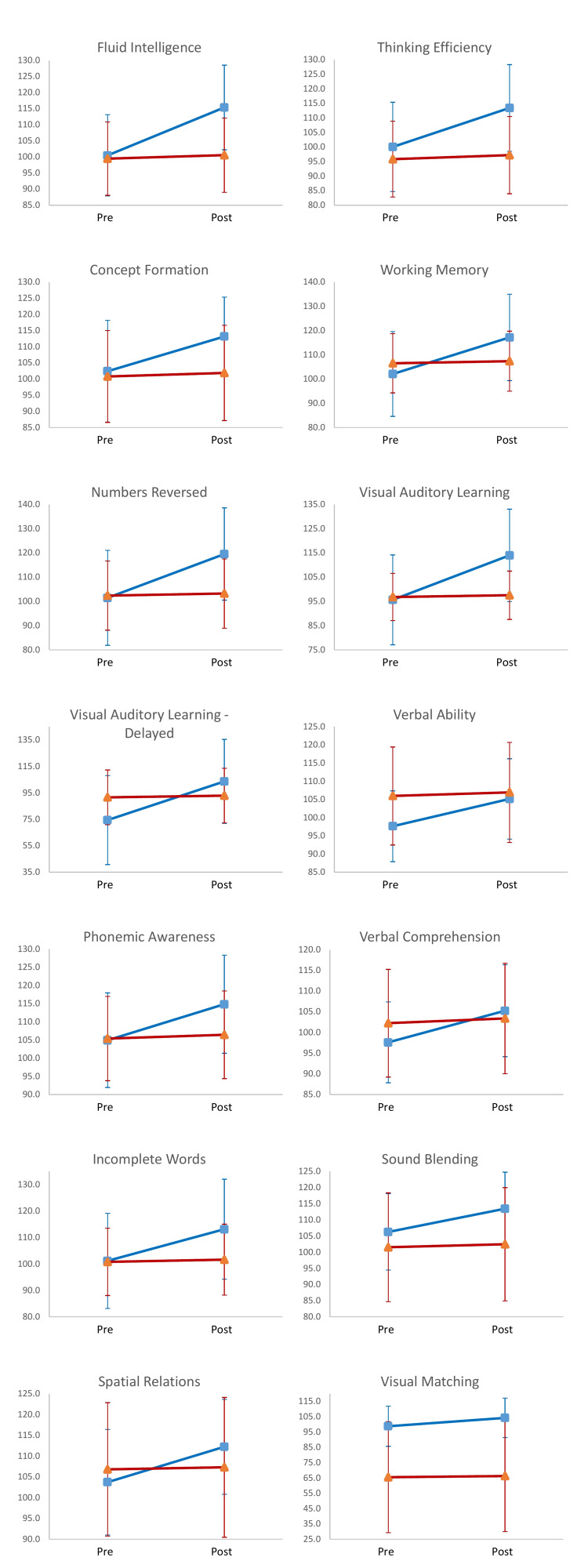
Profile analysis of response to BCI-CRT treatment across 14 domains of the WJII battery. Each subplot shows marginal mean pre- and post-treatment scores for the treatment group (blue, ­n = 200) and non-treatment comparison group (red, n = 121). Bars represent plus/minus one SD of each mean score. BCI: Brain-computer interface; CRT: Cognitive remediation therapy; WJIII: Woodcock-Johnson Test of Cognitive Abilities III.

Table 1 displays mean scores, SDs, and the number of participants between subject groups (treatment, no treatment) for all 14 subtests of the WJIII. After testing the difference for all variables across time between test groups, a significant multivariate effect was found. In addition, the test results revealed a significant multivariate effect for all variables (Table 1). Thus, results imply that participants' measured cognitive abilities in the treatment group increased significantly more across tests administrations than those in the non-treatment group. In Table 2, the eta-squared coefficients are displayed, revealing that between 10% and 53% of the reason why the variables varied across time was due to treatment group status. Figure 1 displays the estimated marginal mean scores for each group across test administrations. The set of changes in pre/post marginal mean scores across each tested WJIII domain constitutes a profile for each treatment group (treated vs. untreated).

**Table 1:** Descriptive statistics of results from pre- and post-tests. Summary of the mean (x̅) and SD (σ) of pre-treatment and post-treatment scores across 14 domains of the Woodcock-Johnson III battery for the treatment group, non-treatment comparison group, and the combined total of all participants. Differences between pre-treatment means and post-treatment means are listed under $\Delta$x̅.

	Treatment (n = 200)					Non-Treatment (n = 121)					Combined (n = 321)				
	Pre		Post			Pre		Post			Pre		Post		
WJIII Domain	x̅	σ	x̅	σ	$\Delta$x̅	x̅	σ	x̅	σ	$\Delta$x̅	x̅	σ	x̅	σ	$\Delta$x̅
Fluid Intelligence	100.5	12.6	115.4	13.1	14.9	99.5	11.4	100.6	11.5	1.1	100.2	12.3	109.5	14.7	9.3
Thinking Efficiency	100.1	15.3	113.4	14.9	13.4	95.8	13.0	97.2	13.3	1.4	98.5	14.6	107.3	16.4	8.8
Concept Formation	102.4	15.7	113.2	12.2	10.7	100.8	14.2	101.9	14.7	1.1	101.9	15.3	108.9	14.3	7.0
Working Memory	102.1	17.5	117.2	17.8	15.1	106.5	12.2	107.4	12.4	0.9	103.8	15.9	113.6	16.7	9.8
Numbers Reversed	101.5	19.6	119.5	19.0	18.1	102.4	14.3	103.2	14.3	0.9	101.9	17.8	113.4	19.1	11.5
Visual-Auditory Learning	95.6	18.5	114.0	19.0	18.3	96.8	9.7	97.5	10.0	0.7	96.1	15.9	108.1	18.0	12.0
Visual-Auditory Learning - Delayed	74.4	33.6	103.6	31.8	29.3	91.6	20.6	92.9	20.7	1.3	81.0	30.6	99.5	28.8	18.5
Verbal Ability	97.6	9.7	105.1	11.0	7.5	106.0	13.5	106.9	13.7	1.0	100.9	12.2	105.8	12.5	4.9
Phonemic Awareness	104.9	13.0	114.8	13.5	9.9	105.4	11.6	106.4	12.0	1.1	105.2	12.5	111.7	13.7	6.5
Verbal Comprehension	97.6	9.8	105.2	11.1	7.7	102.2	13.0	103.4	13.3	1.1	99.4	11.4	104.4	12.0	5.0
Incomplete Words	101.2	18.0	113.1	18.9	12.0	100.8	12.7	101.6	13.4	0.8	101.1	16.2	108.8	17.8	7.7
Sound Blending	106.3	11.8	113.5	11.3	7.2	101.5	16.8	102.5	17.5	0.9	104.6	14.2	109.6	14.8	5.0
Spatial Relations	103.7	12.7	112.2	11.4	8.5	106.8	16.0	107.3	16.8	0.5	104.8	14.1	110.4	13.6	5.6
Visual Matching	98.7	13.1	104.2	12.9	5.5	65.5	36.3	66.2	36.3	0.7	86.4	29.3	90.2	30.5	3.8

**Table 2:** Multivariate analysis of variance. Multivariate analysis shows an inferential response to treatment based on scores across 14 Woodcock-Johnson III battery domains, for subjects in treatment and non-treatment groups. Shown for each domain are Wilk’s Lambda, F statistic, p-value and squared partial eta (Partial $\eta ^2$).

WJIII Domain	Wilk’s Lambda (λ) (1,319)	F	p	Partial $\eta ^2$
Fluid Intelligence	0.463	370.1	<0.001	0.537
Thinking Efficiency	0.651	171.0	<0.001	0.349
Concept Formation	0.774	93.0	<0.001	0.226
Working Memory	0.689	144.1	<0.001	0.311
Numbers Reversed	0.700	137.0	<0.001	0.300
Visual-Auditory Learning	0.688	144.7	<0.001	0.312
Visual-Auditory Learning - Delayed	0.677	152.5	<0.001	0.323
Verbal Ability	0.747	107.8	<0.001	0.253
Phonemic Awareness	0.726	120.6	<0.001	0.274
Verbal Comprehension	0.752	105.2	<0.001	0.248
Incomplete Words	0.808	75.7	<0.001	0.192
Sound Blending	0.834	63.4	<0.001	0.160
Spatial Relations	0.814	73.0	<0.001	0.186
Visual Matching	0.900	35.5	<0.001	0.100

Effects on sobriety and social re-integration

A random set of treatment and non-treatment participants were followed for 18 months to track the integrative effect of the program. At the 18-month follow-up assessment, 89% of the treatment group had maintained sobriety, and 98% had transferred to sober living facilities and maintained an independent residence. Conversely, the sobriety rate of the non-treatment group was 31%, which is consistent with the sobriety rates reported in the literature [2-4]. In addition, the 89% abstinence rate marks a substantial improvement compared with the 20%-40% sobriety rate reported in the literature.

## Discussion

Individuals in recovery exhibit persistent neurophysiological deficits affecting cognitive performance. For example, regarding cognitive control, abstinent cocaine users show reduced metabolism in the left anterior cingulate cortex (ACC) and right dorsolateral prefrontal cortex (DLPFC), including greater activation in the right ACC [16]. The ACC contributes to two essential aspects of executive control: inhibitory control and performance monitoring [16]. Performance monitoring processes include error detection and conflict monitoring, whereas inhibitory control restrains desired behaviors [30-32].

Neuroscience models of cognitive control emphasize that when the ACC detects erroneous or conflicting behavior, a signal is sent to the DLPFC [30-32]. The DLPFC modulates and sustains goal-oriented behaviors by influencing top-down cognitive control, directing behaviors away from incorrect, conflict-causing responses and toward correct, conflict-reducing responses [30-32]. With regard to addiction and sobriety, these monitoring and modulating processes are valuable for detecting hazardous situations or behaviors that increase the likelihood of relapse [33]. Importantly, previous studies have shown that reduced metabolic activity in these brain regions predicts relapse behaviors in both abstinent and active cocaine users [34-36]. In addition, individuals demonstrating healthier ACC activity at the onset of abstinence are less likely to relapse [33-36]. Equally important, performance scores on behavioral monitoring tasks in conjunction with neuroimaging data (using Stroop and decision-making activities known to activate cognitive control neuronal circuits) predict the probability of completing treatment [37,38]. Thus, cognitive control circuits are reliable targets for relapse prediction and neuronal rehabilitation training. The current study posited that similar tasks help evaluate functional changes in cortical circuits that underlie inhibitory control and the action monitoring of abstinence.

In a pilot study of poly-substance users, Gunkelman J and Cripe C used EEG-based neurometrics to identify and establish two joint neural factors observed in most addiction cases [26]. Each factor was considered to represent a separate pathophysiologic drive toward addictive behaviors: a) over-arousal of CNS involving DLPFC disruptions and b) cingulate issues (ACC disruptions and compulsive hyper/hypo foci). After applying EEG phenotype modeling methods [39], the authors derived a standard set of BCI protocols to monitor the EEG responses acquired during CRT training [21,26]. This training targeted executive function and ACC engagement to influence the level of difficulty of the activity [21, 26]. The activities included a collection of modified Stroop activities, go/no-go activities, working memory activities, attention-binding activities, and other executive function activities [21]. Cripe has previously detailed the design and development of these training tools [21, 26]. The present study employed the BCI-monitoring methodology explained earlier with the addition of executive function and working memory activities. These activities aimed to simulate neuro-resilience training by varying cognitive load during training. In addition, the study investigated whether 1) BCI-augmented CRT methods can increase participants' cognitive control abilities and 2) this increase may allow recovering participants to maintain sobriety at higher rates than the 20%-40% treatment average.

## Conclusions

A BCI-augmented CRT treatment method targeted at strengthening executive self-control abilities showed a significant impact on a treatment group's cognitive abilities and sobriety performance compared to untreated control. Comparisons of the pre- and post-treatment results between treated and non-treated participants suggested a causal inferential response to positive treatment effects, suggesting that using a BCI-augmented CRT method increases cognitive control abilities in recovering participants. Furthermore, when considering participants' qualitative sobriety/social reintegration reports, increased abstinence rates in treated versus non-treated participants raise the possibility that increased executive function abilities contribute to a participant's ability to maintain sobriety more effectively than the currently published recovery rates of 20%-40%. Nevertheless, the current results only suggest that BCI-augmented CRT training helps strengthen executive self-control abilities, which might improve sobriety rates. The principal limitations of this study were 1) the fact that it employed a retrospective design and 2) the fact that participants were paid. Follow-up studies that compare BCI plus CRT versus CRT alone versus no treatment or sham BCI study conditions are needed to determine which combination of BCI and CRT treatment methods is most effective.
